# Formula feeding practice and associated factors among mothers with infants 0–6 months of age in Addis Ababa, Ethiopia: a community-based cross-sectional study

**DOI:** 10.1186/s13052-021-01010-x

**Published:** 2021-03-09

**Authors:** Alemnesh Abebe Taye, Wondwosen Asegidew, Mitku Mammo Taderegew, Yonas Girma Bizuwork, Betregiorgis Zegeye

**Affiliations:** 1 Department of Public Health, College of Health Science, Debre Berhan University, Debre Berhan, Ethiopia; 2 Department of Biomedical Sciences, College of Medicine and Health Sciences, Wolkite University, P.O. Box 07, Wolkite, Ethiopia; 3 USAID HIV Control Grant, Addis Ababa, Ethiopia; 4 HaSET Maternal and Child Health Research Program, Shewarobit Field Office, Shewarobit, Ethiopia

**Keywords:** Formula, Breastfeeding, Predictor, Ethiopia

## Abstract

**Background:**

Lack of exclusive breastfeeding during the first half-year of life is an important risk factor for childhood morbidity and mortality. Despite this, less than 40% of infants below 6 months are exclusively breastfed worldwide. This is because breastfeeding is declining and being replaced by formula feeding. Nowaday, formula feeding has become a more common practice in urban communities of developing countries. However, relatively little information is available regarding formula feeding practice and its associated factors in Ethiopia, particularly in Addis Ababa. Hence, this study was aimed at assessing the prevalence of formula feeding practice and its associated factors among mothers of an infant aged 0–6 months in Addis Ababa, Ethiopia.

**Methods:**

A community-based cross-sectional study was conducted from April-1 to May 30/2020 among 494 mothers with infants 0–6 months of age. Data were collected using a pre-tested structured questionnaire. Data were entered and cleaned by using Epi data version 3.1 and analysed by SPSS software version 25. Then data were processed by using descriptive analysis, including frequency distribution, and summary measures. The degree of association was assessed using binary logistic regression analysis. *P*-value < 0.05 was considered statistically significant.

**Result:**

The prevalence of formula feeding and pre-lacteal feeding practice was 46.2 and 34.4%, respectively. Educational status with a diploma and above (AOR = 3.09, 95%CI: 1.56–6.14), delivery by cesarean section (AOR = 6.13, 95%CI: 4.01–9.37), pre-lacteal feeding practice (AOR = 7.61, 95%CI: 4.11–11.06), and delayed initiation of breastfeeding (after 1 h to 1 day (AOR = 3.43, 95% CI: 1.59–7.40), after 1 day to 3 days (AOR = 3.71, 95% CI: 1.51–9.41), and after 3 days (AOR = 5.41, 95% CI: 2.15–13.60)) were significantly associated with formula feeding practice.

**Conclusions:**

Nearly half of the participants were practiced formula-feeding for their infant. Educational status of mothers, the timing of initiation of breastfeeding, delivery by cesarean section, and pre-lacteal feeding practice were significantly associated with formula feeding practice. Therefore, early initiation of breastfeeding, educating mothers about the risks associated with pre-lacteal feeding, and supporting mothers who gave birth by cesarean section for exclusive breastfeeding should be encouraged at the community and institutional levels.

**Supplementary Information:**

The online version contains supplementary material available at 10.1186/s13052-021-01010-x.

## Background

Infants are in a state of rapid growth and development; the growth rate is most rapid during the first 4 to 6 months of life. Optimal infant and young child feeding practices rank among the most effective interventions to improve child health [1, 2].

The World Health Organization (WHO) [3] and the American Academy of Pediatrics (AAP) [4] recommends exclusive breastfeeding for the first 6 months of life with a continuation of breastfeeding while gradually introducing solid foods into the infant’s diet for 1 year or longer as mutually desired by mother and infant. In 2016, the United Nations (UN) Office of the high commissioner of human rights declared that breastfeeding is a human rights issue for both mothers and children and should be protected and promoted for the benefit of both. The introduction of local, nutrient-rich complementary foods thereafter with continued breastfeeding for 2 years of age or beyond is recommended [5, 6].

Despite, the recommendations that babies should be exclusively breastfed for the first 6 months, less than 40% of infants below this age are exclusively breastfeeding worldwide. This is due to the impacts of globalization, increasing availability of formula milk in the supermarket, and promotion of formula milk by advertising on different media, the proportion, and duration of breastfeeding are declining and being replaced by formula feeding.

Lack of breastfeeding and especially lack of exclusive breastfeeding during the first half-year of life are important risk factors for infant and childhood morbidity and mortality [7, 8]. United Nations international children’s emergency fund (UNICEF) states that the provision of supplemental formulas can increase infant mortality by as much as 25 times [9]. Infants on formula feeding are not only deprived of the benefits of breast milk but also to be affected by acute respiratory infections (ARI), otitis media, allergies, gastroenteritis, diarrhea, pneumonia, diabetes mellitus, decreased cognitive development, increase risk of obesity, and sudden infant death syndrome [2, 8–10].

Formula feeding has become a common practice in developed countries and urban communities in developing countries. Today there is a shift from exclusive breastfeeding practice towards the introduction of bottle-feeding. The increasing incidence of bottle feeding in developing countries particularly in Africa reflects the absorption of the western way of life [11].

Starting from 2016, the Ethiopian government has implemented several directives such as the “Infant Formula and Follow-up Formula Directive No. 30/2016” and the “Food Advertisement Directive 33/2016” to encourage breastfeeding by restricting the promotion of formula feeding practice [12]. However, the proportions of mothers who still breast-feed their child are considerably low particularly among women of the capital city, Addis Ababa [13]. According to the Ethiopian Demographic and Health Survey (EDHS) 2016 report, only 58% of infants less than 6 months were exclusively breastfed [14]. Formula feeding was 30% among the age of up to 1 month, it was 45% between two and 3 months and it increased to 68% in the infants from four to 5 months [2].

Nowadays there are different mechanisms applying in formula milk promotion by advertising on different media including by training health care providers. Due to these, intervention should be done to promote exclusive breastfeeding than infant formula at international and national levels especially in urban communities of Ethiopia. Hence, the study was conducted to assess the formula feeding practice and its associated factors among mothers with infants aged 0–6 months in Addis Ababa city, the capital city of Ethiopia.

The finding of this study will provide relevant updated information regarding the formula feeding practice and its associated factors, which is helpful for policymakers and other stakeholders to develop appropriate strategies and interventions for promoting and maintaining exclusive breastfeeding practices for the first 6 months of an infant’s life. It is also hoped that the study will provide baseline data for further research investigation on the area of this study.

## Methods

### Study setting and design

A community-based cross-sectional study was conducted in Addis Ababa, the capital city of Ethiopia from April 1 to May 30/2020. Addis Ababa city holds a total area of 527 km^2^ and contains 10 sub-cities and 116 woredas. The city has an estimated population of 4,793,699 and each woreda contained an estimated 479,370 populations on average [15].

### Study population and sampling techniques

All mothers who had infants less than 6 months old and lives at randomly selected woreda were eligible for the study whereas mothers, who were critically ill or unable to respond due to serious illness during the data collection period and no other family members living with her, were excluded from the study.

The required sample size was determined using a single population proportion formula by taking the proportion of formula feeding as 68% [2], 95% confidence interval, 5% level of precision, and a design effect of 1.5. Hence, the final sample size was 500.

### Sampling procedures

A multistage sampling technique was used for selecting the study participants. Initially, Gulele, Arada, and Lideta sub-cities (each of which contains 10 woredas) were randomly selected. Then using a simple random sampling technique three woredas were selected from each selected sub-city. Then, the number of mothers who were selected from each selected woreda was determined proportionally. Finally, to select the study participants from each selected woreda, a systematic random sampling technique was used by using the list of mothers from the Epi registration book from each woreda health office (Additional file 1: Fig. S1).

### Data collection procedures and operational definitions

Data were collected by using a pre-tested structured questionnaire which was taken from previously published literature on a similar title in which the cultural and socioeconomic characteristics of study participants were similar to the target population of this study [2]. The questionnaire consists of socio-demography characteristics, maternal health service utilization, and infant-related characteristics. Data were collected at the household level from mothers with infants 0–6 months of age and who resides at selected woreda in Gulele, Arada, and Lideta sub-cities. The questionnaire was initially prepared in English and translated to Amharic (local language) and then back to English with an expert who has a good ability of the two languages to maintain its consistency. Then the final Amharic form of the questionnaire was used to collect the data.

Prior to the actual data collection, the questionnaire was pretested on 10% (50) of the sample size in woreda 03 under Yeka sub-city which was not part of the actual data collection area. Based on the pre-test some modifications have been done to the questionnaire.

Six data collectors (urban health extension workers) and three supervisors (public health professionals) were recruited. One day of training was given for data collectors and supervisors by the investigators on the objectives of the study, data collection procedures, data collection tools, and confidentiality of information. The principal investigator coordinates the overall process of data collection and the activity of the whole study. The data collection procedures were checked frequently through supervision by the investigators and supervisors for its consistency. Moreover, the collected data were checked daily to safeguard its completeness.

In this study, the formula-feeding practice was defined as a positive answer to the question “Did you fed your child any formula feeding as a substitute or supplement to breastfeeding?” Pre-lacteal feeding is also assessed by asking “What did you start to feed your child the day he/she has born?” The mothers who answered “other than breast milk” to the question were considered a mother who practiced pre-lacteal feeding. The infant’s birth weight was recorded by reviewing the registration book in the delivery room from the health facility in which the mother was delivered.

### Operational definition

**Birth weight of infants:** Low birth weight (birth weight <2500 g); normal birth weight (2500 g ≤ birth weight <4000 g); and over birth weight (birth weight ≥ 4000 g) [16].

**Formula feeding practice:** Feeding of an infant less than 6 months old with formula food or bottle feeding as a substitute for or supplement to breastfeeding [2].

**Pre-lacteal feeding:** Feeding of an infant with any fluid or semisolid food before the mother has begun to breastfeed [17].

**Timely initiation of breast milk:** Initiation of breast milk within 1 hour of delivery [18].

**Woreda:** The third-level administrative divisions of Ethiopia which further subdivided into several kebele or neighborhood associations.

### Data processing and analysis

The collected data were checked manually for its completeness and consistency. Then the data were coded and entered into Epi-data version 3.1 and double enters by another person for consistency. Finally, the data were exported to SPSS-version 25 for further analysis. Multicolinearity among selected independent variables was checked via “Variance inflation factor (VIF) and Tolerance” and all variables were with less than 3 and above 0.2 VIF and tolerance, respectively. The data were processed by using descriptive analysis, including frequency distribution, and summary measures. Categorical variables were stated as number (percentage) whereas the continuous data as means ± standard deviation (SD).

To determine the independently associated variables, associations were investigated using binary logistic regression analysis. All independent variables with *p*-value < 0.25 in the unadjusted model were selected as a candidate for multivariable analysis to control the effect of confounding variables. Model fitting at the “hosemer and lemeshew test” was done to check model fitness at a non-significant level in multivariable analysis. Then the degree of associations was expressed by using the odds ratio (ORs) with 95% CI. *P*-value < 0.05 was considered statistically significant.

## Results

### Socio-demographic characteristics of the study participants

A total of 500 mothers were invited and 494 of them volunteered to participate in the study making the response rate 98.8%. The mean age (±SD) of the respondents was 30.11 (±5.73) years. More than half (60.1%) of the respondents were in the age group 25–34 years. The majority (96.2%) of participants were married, 198 (40.1%) were housewives, and 164 (33.2%) were with educational status of degree and above (Table 1).

**Table 1:** Socio-demographic characteristics of mothers having infants less than 6 months old, in Addis Ababa city, Ethiopia, 2020 (*N* = 494)

Variables	Category	Frequency (*N* = 494)	Percent (%)
Age (years)	<25	64	13.0
	25–34	297	60.1
	≥35	133	26.9
Educational status	Primary	77	15.6
	Secondary	112	22.7
	Certificate/diploma	141	28.5
	Degree and above	164	33.2
Occupational status	House wife	198	40.1
	Private employee	169	34.2
	Government employee	110	22.3
	Others^a^	17	3.4
Current marital Status	Married	475	96.2
	Single/Divorced	19	3.8

Note: ^a^Student and unemployed

### Health service utilization of mothers and infant related characteristics

Among the total of the respondents, 298 (60.3%) were multipara, 489 (99.0%) were received antenatal care (ANC) services during pregnancy, and all participants gave birth at health facilities. The majority (93.5%) of the participants received postnatal care (PNC) counseling about breastfeeding and complementary feeding. Regarding infants related characteristics, more than half (56.3%) of the infants were in the age group 4–6 months, 422 (85.4%) had normal birth weight, and 330 (66.8%) started breastfeeding within 1 h of delivery (Table 2).

**Table 2:** Health service utilization among mothers having infants less than 6 months old, and infants related characteristics in Addis Ababa, Ethiopia, 2020 (*n* = 494)

Variables	Categories	Frequency	Percent
Parity	Prim- Para	196	39.7
	Multi-para	298	60.3
ANC follow-up	No	5	1.0
	Yes	489	99.0
Number of ANC visit	<3	49	10.0
	≥ 3	440	90.0
Place of ANC follow-up	Public health institution	261	53.4
	Private health institution	228	46.6
Place of delivery	Public health institution	251	50.8
	Private health institution	243	49.2
Mode of delivery	Spontaneous/vaginal	294	59.5
	Cesarean section	200	40.5
Age of child (months)	< 2	166	33.6
	2–3	50	10.1
	4–6	278	56.3
Sex of the child	Male	284	57.5
	Female	210	42.5
Weight of the child	Under weight	32	6.5
	Normal weight	422	85.4
	Over weight	40	8.1
Timely initiation of breast feeding	Within 1 h	330	66.8
	1 h -1 day	75	15.2
	1 day-3 days	69	14.0
	Above 3 days	20	4.0
Pre-lacteal feeding	Yes	170	34.4
	No	324	65.6

*ANC* Antenatal care

### Formula feeding practice among the study participants

**Fig. 1:**
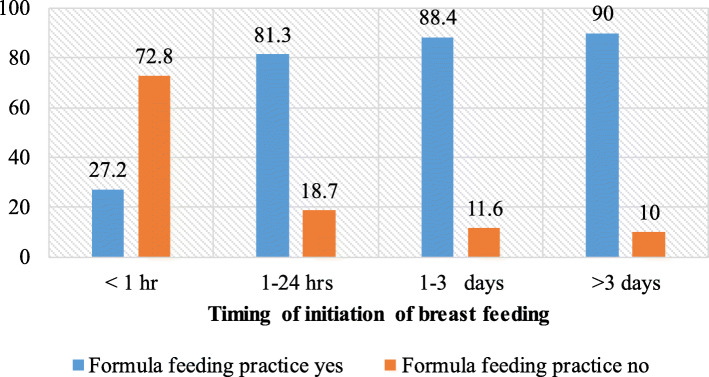
Percentage of formula feeding practice based on timing of initiation of breastfeeding among mothers having infants less than 6 months old in Addis Ababa, Ethiopia, 2020

In this study, the prevalence of formula feeding was 46.2% (95% CI: 41.3–49.7%). Among the infants with formula feeding, more than half (54.4%) were males. The prevalence of formula feeding practice was higher among infants aged 2–3 months (52%), low birth weight 18 (56.3%), delivery by cesarean section 143 (71.5%), infants with pre-lacteal feeding 145 (85.3%), and infants who started breastfeeding after 3 days of delivery 16 (80%) (Fig. 1).

### Awareness of mothers towards infant formula feeding

Among the study participants, 412 (83.4%) have heard about infant formula feeding. The most frequent source of information for formula feeding practice was mass media, i.e. television and radio 194 (47.1%); other sources were relatives 111 (26.9), health facility staff 85 (20.6), and supermarket/pharmacy staff 22 (5.4%). The most common reported reason behind infant formula feeding practice among mothers was insufficient breast milk production 181(79.4%), a mother being sick 36 (15.8%), and a baby being sick 11 (4.8%).

### Factors associated with formula feeding practice

On a multivariable logistic regression analysis educational status of mothers with a diploma and above (AOR =3.09, 95% CI: 1.56–6.14), the timing of initiation of breastfeeding (after 1 h to 1 day (AOR =3.43, 95% CI: 1.59–7.40), after 1 day to 3 days (AOR =3.71, 95% CI: 1.51–9.41), and after 3 days (AOR =5.41, 95% CI: 2.15–13.60)), delivery by cesarean section (AOR =6.13, 95% CI: 4.01–9.37), and presence of pre-lacteal feeding (AOR =7.61, 95% CI: 4.11–11.06) were significantly associated with formula feeding practice (Table 3).

**Table 3:** Binary logistic regression analyses of factors associated with formula feeding among mothers with infants less than 6 months old in Addis Ababa, Ethiopia, 2020 (*N* = 494)

Variables	Formula feeding practice			
	Yes (%)	No (%)	COR (95% CI)	AOR (95% CI)
Education				
Primary school	17 (22.1)	60 (77.9)	1	1
Secondary school	39 (34.8)	73 (65.2)	1.89 (1.04–3.61)*	1.77 (0.93–3.36)
Diploma & above	172 (56.4)	133 (43.6)	4.56 (2.28–7.46)*	3.09 (1.56–6.14)*
Occupation				
House wife	74 (37.4)	124 (62.6)	1	1
Government	56 (50.9)	54 (49.1)	1.74 (1.14–2.94)*	1.30 (0.73–2.33)
Private and others	98 (52.7)	88 (47.3)	1.87 (1.25–2.83)*	1.59 (.98–2.56)
Marital status				
Married	224 (47.2)	251 (52.8)	3.35 (1.13–10.53)*	2.79 (0.84–9.33)
Single/divorced	4 (21.1)	15 (78.9)	1	1
Place of ANC				
Public	100 (38.3)	161 (61.7)	1	1
Private	128 (56.1)	100 (43.9)	2.06 (0.51–4.41)	1.60 (0.83–3.09)
Number of ANC visit				
< 3	32 (65.3)	17 (34.7)	1	1
≥3	196 (44.5)	244 (55.5)	0.43 (0.01–0.437)*	0.60 (0.09–4.26)
Place of delivery				
Public institutions	95 (37.8)	156 (62.2)	1	1
Private institutions	133 (54.7)	110 (45.3)	1.99 (1.87–2.85)*	1.08 (0.45–1.72)
Mode of delivery				
Normal/vaginal	85 (28.9)	209 (71.1)	1	1
Cesarean section	143 (71.5)	57 (28.5)	6.17 (4.35–9.09)*	6.13 (4.01–9.37)*
Timing of initiation of breastfeeding				
Within 1 h	110 (33.3)	220 (66.7)	1	1
After 1 h to 1 day	50 (66.7)	25 (33.3)	4.00 (6.19–21.86)*	3.43 (1.59–7.40)*
After 1 day to 3 days	52 (75.4)	17 (24.6)	6.12 (9.37–44.27)*	3.71 (1.51–9.41)*
After 3 days	16 (80.0)	4 (20.0)	8.00 (5.46–25.71)*	5.41 (2.15–13.60)*
Pre-lacteal feeding				
Yes	145 (85.3)	25 (14.7)	11.84 (7.12–25.59)*	7.61 (4.11–11.06)*
No	83 (25.6)	241 (74.4)	1	1
Age of the child				
< 2 month	63 (38.0)	103 (62.0)	1	1
2–3 months	26 (52.0)	24 (48.0)	1.77 (1.62–2.77)*	2.17 (0.98–3.14)
4–6 months	139 (50.0)	139 (50.0)	1.63 (1.29–3.11)*	1.92 (0.74–3.09)

*Significant at a *p*-value of <0.05, *BF* Breastfeeding, *ANC* Antenatal care, *C/S* Cesarean section

## Discussion

This study has assessed the prevalence of formula feeding practice and associated factors among mothers with infants 0–6 months of age. Accordingly, the study found that 46.2% of mothers in the study area used formula feeding, 34.4% fed their infant pre-lacteal fluid. It was also found that the educational status of mothers, timing of initiation breastfeeding, pre-lacteal feeding, and delivery by cesarean section were significantly associated with formula feeding practices.

The prevalence of formula feeding practice in this study was similar to the EDHS 2011 result [19], the study in Eastern Ethiopia [20], and Jimma, Southwest Ethiopia [2]. However, this was higher than the findings from the studies conducted at Holeta (19.6%) [21], Gozamin, Northwest Ethiopia [22], Bodity, Southern Ethiopia [23], and Shashemene (20.9%) [24], but lower than the study conducted in Agaro, Southwest Ethiopia [25], and Harar [18]. The reasons for this difference in the prevalence of formula feeding practice may be due to variations in the study setting, sociocultural characteristics of participants, employment status of participants, availability of health care services, and health service utilization. Due to the urban area of the study, the participants in this study may be more likely to be government employees as compared to the study elsewhere in the rural area and hence the prevalence of formula feeding practice may be higher.

Concerning factors associated with formula feeding, this study found that mothers with an educational level of college diploma and above were more likely to practice formula feed for their child as compared to those with primary education and below. While this finding is comparable with similar studies conducted in Agaro, Southwest Ethiopia [25], Jimma, Southwest Ethiopia [2], and Cameroon [26], it is contrary to a study conducted in Indonesia [9] where educated mothers were 39% less likely to formula feed their infants. The reason for this discrepancy may be related to the methodology of the studies, classification of educational status, or the cultural differences of the study subjects.

Mode of delivery was also significantly associated with the formula-feeding practice, as mothers who gave birth by cesarean section were more likely to feed formula as compared to those who gave birth vaginally. This is in agreement with a similar study conducted in Bahirdar [27], Addis Ababa [28], and Egypt [29] where cesarean delivery was significantly associated with formula feeding practice. This may be due to post-operative conditions, as mothers with cesarean sections were less likely to have had skin-to-skin contact with their infants and felt fatigued and less relaxed after birth in the delivery room. This causes improper breast stimulation and emptying which in turn reduce maternal milk secretion and cause the introduction of formula feeding.

Contrary to this finding, another study conducted in a group of mothers in Egypt showed that cesarean delivery was 41.9% less likely associated with formula feeding practice [11]. The study in Debre Tabor town, Northwest Ethiopia, also found that mothers, who gave birth vaginally, were two times more likely to practice formula feeding than mothers who gave birth with cesarean section [30]. The reason for this discrepancy may be related to the methodology, period of the study, classification of mothers who delivered by cesarean section (mixed feeding group and exclusive formula feeding group), and the cultural differences of the study subjects.

Another predictor for formula feeding practice in this study was the timely initiation of breast milk. Mothers who initiated breastfeeding after 1 hour to 1 day of delivery were three times more likely to practice formula feeding compared to those who initiated breastfeeding within 1 hour. Similarly, mothers who initiated breastfeeding after 1 day up to 3 days and after 3 days were approximately four and five times more likely to practice formula feeding compared to those who initiated breastfeeding within 1 h, respectively. This finding was in agreement with studies from Hossana [31], Afar region [32], and Offa district, Southern Ethiopia [17]. The possible reason for this might be due to the fact that mothers who practiced early initiation of breastfeeding may have relatively good knowledge, attitude, and practice towards exclusive breastfeeding and also may have a better understanding about the risk of formula feeding for infants under the age of 6 months. When the time interval between delivery and initiation of breastfeeding is increases, there is a chance for the initiation of pre-lacteal feeding practice which in turn leads to decreased newborn-mother bonding and then inadequate maternal breast milk secretion.

It was also likely that the difference in the practice of formula-feeding may result from the difference in pre-lacteal feeding. Compared to mothers who didn’t give pre-lacteal feeds for their infant, mothers who gave pre-lacteal feeding were eight times more likely to practice formula-feeding. This finding is consistent with the findings of the studies in Raya Kobo district, North Eastern Ethiopia [33], Debre Markos [34], and Motta, Northwest Ethiopia [35]. This may be because pre-lacteal feeding cause delays in the need of an infant’s immediate breastfeeding and decreases the infant’s suckling and breast stimulation activity which in turn leads to inadequate milk production. The decrease in milk production will lead mothers to introduce supplementary foods for their infants and the addition of this supplementary formula may cause more and more reduction in milk production.

The study has some limitations. Due to the cross-sectional nature of the study design, the cause and effect relationship of events cannot be ascertained; moreover, as data were collected based on the mother’s perspective and self-reports rather than the practice being observed, the recall and social desirability biases may represent further limitations of the study.

## Conclusion

Nearly half of the participants had formula-feeding practiced and one-third of participants were given pre-lacteal feeding for their infants. Educational status of mothers, the timing of initiation of breastfeeding, mode of delivery, and pre-lacteal feeding practice were found to be significantly and independently associated with formula feeding practice. Therefore, early initiation of breastfeeding, educating mothers about the risks associated with pre-lacteal feeding, and supporting mothers who gave birth by cesarean section for exclusive breastfeeding should be encouraged at the community and institutional levels.

## Supplementary Information

**Additional file 1: Fig. S1**: Schematic representation sampling procedure for assessing formula feeding practice in Addis Ababa city, 2020

## Data Availability

The datasets used and/or analyzed during the current study are available from the corresponding author on reasonable request.

## Abbreviations

AAP: American academy of pediatrics
ANC: Antenatal care
AOR: Adjusted odds ratio
ART: Acute respiratory infections
COR: Crude odds ratio
EBF: Exclusive breastfeeding
EDHS: Ethiopian demographic and health survey
IRB: Institutional review board
PNC: Postnatal care
WHO: World health Organization
