# Supplementary figures and images for: Formula feeding practice and associated factors among mothers with infants 0–6 months of age in Addis Ababa, Ethiopia: a community-based cross-sectional study
Source: Ital J Pediatr. 2021 Mar 9;47:55. doi: 10.1186/s13052-021-01010-x (PMC7941680; doi:10.1186/s13052-021-01010-x)

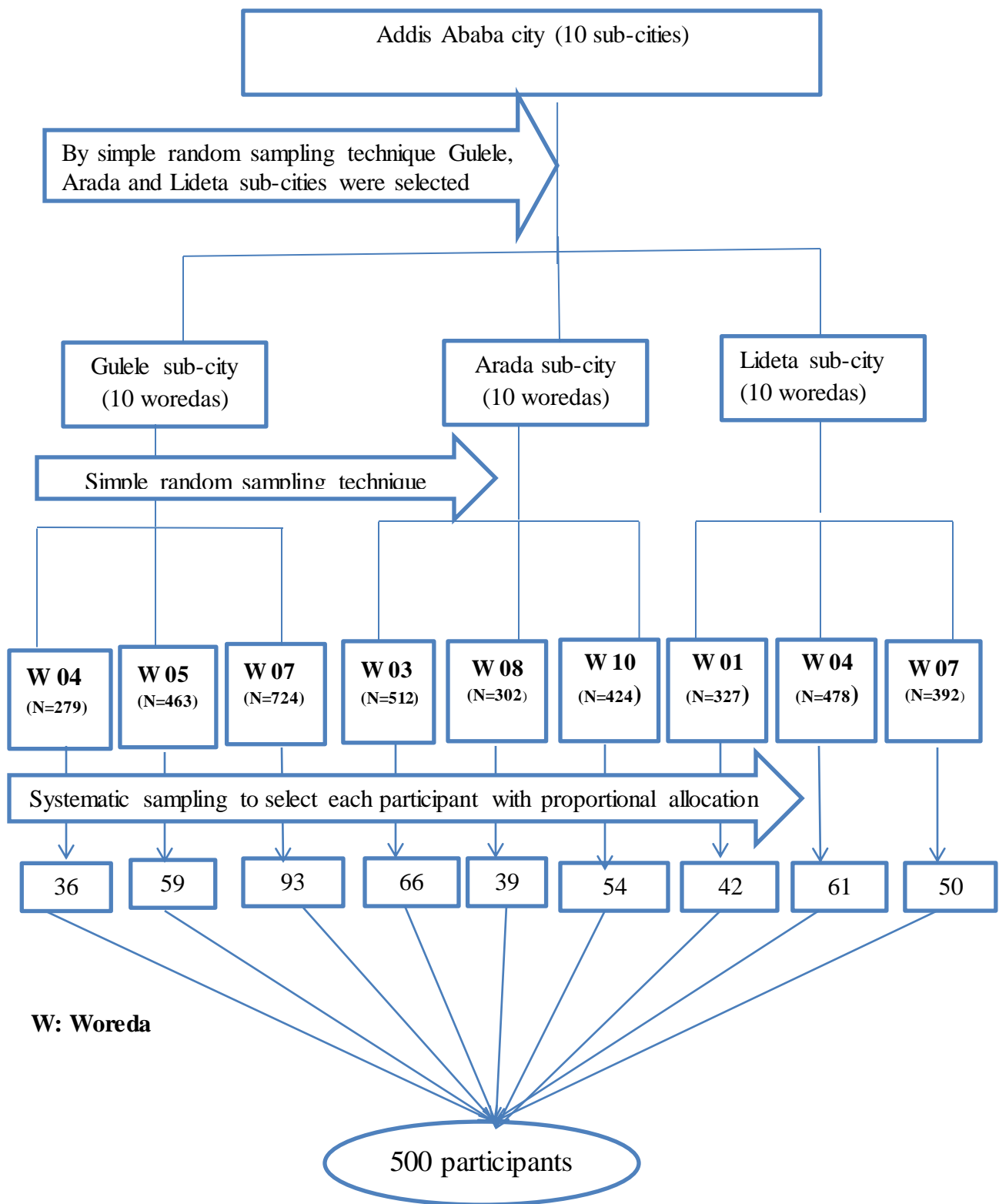

Supplement: Supplementary file 1 — Additional file 1: Fig. S1: Schematic representation sampling procedure for assessing formula feeding practice in Addis Ababa city, 2020 [file 13052_2021_1010_MOESM1_ESM.pdf]
